# Association of British Clinical Diabetologists and UK Kidney Association Joint Clinical Practice Guidelines for the Pharmacological Management of Hyperglycemia in Adults With Type 2 Diabetes Mellitus and CKD

**DOI:** 10.1016/j.ekir.2025.07.028

**Published:** 2025-07-31

**Authors:** Janaka Karalliedde, Kieran McCafferty, Peter Winocour, Tahseen A. Chowdhury, Naresh Kanumilli, Parijat De, Andrew H. Frankel, Ciara Doherty, Nicola Milne, Rosa M. Montero, Eirini Loudaki, Debasish Banerjee, Ritwika Mallik, Adnan Sharif, Sagen Zac-Varghese, Srikanth Bellary, Gabrielle Goldet, Ketan Dhatariya, Stephen C. Bain, Indranil Dasgupta

**Affiliations:** 1 School of Cardiovascular and Metabolic Medicine and Sciences, King’s College London, London, UK; 2 Department of Nephrology, Queen Mary University London and Barts Health NHS Trust, London, UK; 3 Department of Diabetes, East and North Herts NHS Trust, Hertfordshire, UK; 4 Department of Diabetes, Royal London Hospital, London, UK; 5 Northenden Group Practice, Manchester, UK; 6 Department of Diabetes, City Hospital, Birmingham, UK; 7 Department of Nephrology, Imperial College Healthcare NHS Trust, London, UK; 8 Department of Diabetes, Guy’s and St Thomas’ Hospital London, London, UK; 9 Brooklands and Northenden Primary Care Network, Manchester, UK; 10 Department of Nephrology, St George’s Hospital, London, UK; 11 Department of Nephrology, King’s College Hospital, London, UK; 12 Department of Nephrology, St George’s University Hospitals NHS Foundation Trust, London, UK; 13 City St George’s University of London, London, UK; 14 Department of Diabetes, University College London NHS Trust London, London, UK; 15 Department of Nephrology, University Hospitals Birmingham, Birmingham, UK; 16 Department of Diabetes, Aston University, Birmingham, UK; 17 Department of Diabetes, Norfolk and Norwich University Hospitals NHS Foundation Trust, Norwich, UK; 18 Norwich Medical School, University of East Anglia, Norwich, UK; 19 Department of Diabetes, Swansea University, Swansea, UK; 20 Department of Nephrology, Heartlands Hospital, Birmingham, UK; 21 Warwick Medical School, University of Warwick, Coventry, UK

## Abstract

A growing and significant number of people with diabetes develop chronic kidney disease (CKD), and diabetes-related CKD is a leading cause of end-stage kidney disease (ESKD). People with diabetes and CKD have high morbidity and mortality, predominantly related to cardiovascular disease (CVD).

Hyperglycemia and hypertension are modifiable risk factors to prevent the onset and progression of CKD and related CVD. Recent clinical trials of people with type 2 diabetes mellitus (T2DM) and CKD have demonstrated reduction in composite kidney end point events (significant decline in kidney function, need for kidney replacement therapy, and kidney-related death) and cardiovascular risk with sodium-glucose cotransporter 2 (SGLT-2) inhibitors, nonsteroidal mineralocorticoid receptor antagonists (nsMRAs) and glucagon-like peptide 1 (GLP-1) receptor agonists (RAs).

The Association of British Clinical Diabetologists and UK Kidney Association Diabetic Kidney Disease Clinical Speciality Group have previously undertaken a narrative review and critical appraisal of the available evidence to inform clinical practice guidelines for the pharmacological management of hyperglycemia in adults with T2DM and CKD. This 2025 abbreviated updated guidance by a multidisciplinary group of health care professionals from primary and secondary care settings summarizes the key recommendations, clinical considerations and recent evidence that has implications for clinical practice for health care professionals who treat people with T2DM and CKD.

### Background

International clinical guidelines advocate a multifactorial approach to the management of CKD secondary to T2DM.^1^^,^^2^ A fundamental and foundational component of this approach is lifestyle interventions, such as a healthy diet and physical exercise to achieve weight loss, and smoking cessation. In parallel, pharmacological treatment and management of glucose, blood pressure, and lipids are recommended. For many decades, renin-angiotensin system (RAS) inhibition was the only proven effective treatment for slowing the progression of CKD, and the effect of this class of agents was independent of their antihypertensive properties.^3^^,^^4^^,^^5^^,^^6^

Of note, these kidney end point trials were not statistically powered sufficiently to draw conclusions from their cardiovascular end points. Although RAS inhibition demonstrated a significant reduction in kidney end points, a significant residual risk of kidney disease progression^5^, ^6^, ^7^ was observed with > 30% of participants progressing to the primary kidney end point^8^^,^^9^

SGLT-2 inhibitors were originally licensed as glucose-lowering agents in T2DM. However, in cardiovascular outcomes trials that were designed primarily to establish the CVD safety of this class, a reduction in kidney events explored as secondary outcomes was observed.^10^

Subsequently clinical trials with dedicated primary kidney end points demonstrated significant reductions in kidney disease events and associated CVD burden, including heart failure.^11^ Following on from the emergence of SGLT-2 inhibition for CKD, nsMRAs, and GLP-1 RAs have also recently demonstrated kidney and cardiovascular benefits effects in people with T2DM and CKD.^11^^,^^12^

This article summarizes the key recommendations for clinical care practice from a group of multidisciplinary primary (community) and secondary care–based health care professions involved in the care of people with T2DM and CKD. The definitions of the evidence grades are presented in Supplementary Figure S1. These recommendations are based on a narrative review of the Cochrane Library, PubMed/MEDLINE, Google Scholar, and Embase carried out initially between October 2013 and December 2020 and further review carried out from December 2020 and February 2025 for the current update, using the following keywords: type 2 diabetes, CKD, diabetic nephropathy, hyperglycemia, hypertension, SGLT-2 inhibitors, non-steroidal mineralocorticoid antagonists, pioglitazone, dipeptidyl peptidase-4 (DPP-4) inhibitors, GLP-1 receptor agonists/analogues and antidiabetic medications, kidney failure, and end-stage kidney disease.

### Glycemic Targets for the Prevention and Management of CKD in People With Diabetes

The management of diabetes is predicated on the basis of reducing hyperglycemia to improve osmotic symptoms, with supportive evidence that this will prevent the onset, and slow down progression of kidney and vascular complications over time.^1^

The precise level of glycemic control that delivers optimal benefit remains contentious because, inevitably, the individualized approach to care and the evidence base from different cohorts do not allow clear extrapolation. The glycemic management of people with type 1 diabetes and type 2 diabetes and the respective kidney benefits require separate considerations, which in part reflects the different evidence base and lifetime risks of complications with the greater risk for hypoglycemia that arises when several concurrent therapies are used alongside insulin as kidney function deteriorates.^1^

The risks of hypoglycemia are greater in people with diabetes and CKD especially if people are on insulin treatment, sulfonylurea or glinides.^1^ Individualized pragmatic glycemic goals that balance the benefits and risks of intensive glucose lowering in people with diabetes and CKD, patient education on hypoglycemia avoidance, and self-management are needed. In addition, the risk-benefit equation of tighter glycemic control for kidney and vascular complications alters as CKD progresses.

There has been an important shift in emphasis in recent international guidance for people with T2DM, with a specific emphasis on the selection of medications, where independent of their glucose-lowering effects, those with evidence base for cardio-kidney protection should be prioritised.^1^^,^^13^

### Glycated Hemoglobin Targets for People who Have T2DM and CKD

Individualized glycated hemoglobin (HbA1c) targets should be applied in the management of people with CKD. We therefore recommend that individualized HbA1c targets should be applied in the management of people with T2DM and CKD, using the levels suggested in Table 1. These target ranges are based on the opinion of the writing committee because there is limited high grade evidence in people with CKD.

**Table 1:** Proposed glycemic targets in people with type 2 diabetes mellitus and CKD

Glycemic target range	CKD stage and albuminuria	Age
48–58 mmol/mol (6.5%–7.5%)^a^ Aim for < 53 mmol/mol (7%)^b^	CKD stages 1–2	People who are aged < 40 yrs Diet controlled at any age^c^
53–58 mmol/mol (7%–7.5%)	CKD stages 3–4 May be appropriate with a GLP-1 and/ or SGLT-2 inhibitor-based treatment regime without insulin^d^	Any age
58–68 mmol/mol (7.5%–8.5 %)	CKD stages 3–4^a^ and those with CKD stage 5 who are on dialysis.^a^ Especially in people with albuminuria who are on an insulin-based regime^d^	Any age

CKD, chronic kidney disease; HbA1c, glycated hemoglobin; GLP-1, glucagon-like peptide 1; SGLT-2, sodium-glucose cotransporter 2.

These recommendations are based on the opinion of the writing group because this is limited high grade evidence in CKD.

a Confirmatory blood glucose or continuous glucose monitoring (CGM) if concern of hypoglycemia and/or anemia as HbA1c will be less reliable in advanced CKD stage 4 or 5.

b 42–48 mmol/mol (6%–6.5%) without hypoglycemia.

c Over 20% of people with CKD (especially older people aged >75 yrs) solely dietary controlled can have HbA1c.

d Recognition of cardio-kidney benefits with SGLT-2 inhibitors and GLP-1 analogue therapy.

At present, it would be prudent to consider an HbA1c target of 58 mmol/mol (7.5%) for most people with CKD if their glucose-lowering therapies include insulin and a target of up to 68 mmol/mol (8.5%) in frail people with more advanced CKD (stage 4 and above). In people with more advanced CKD, particularly with estimated glomerular filtration rate (eGFR) < 30 ml/min per 1.73 m^2^, HbA1c has more limitations and we would advise monitoring of capillary blood glucose or continuous glucose monitoring to aid clinical decision and treatment choice.^14^^,^^15^

It remains unclear with no evidence to date whether it is appropriate and or safe to have a lower glycemic HbA1c target of below 52 mmol/mol (6.9%) for reduction in CKD onset or progression with novel agents such as GLP-1 RAs, dual GLP-1 RA and glucose-dependent insulinotropic polypeptide (GIP) agonist, or SGLT-2 inhibitors, which have lower burden of hypoglycemia. From the current evidence, there is no basis to seek HbA1c values < 53 mmol/mol (7%) in older people with T2DM and CKD with medication.

### Kidney Function Measurements in Determining Medication Dosages in Diabetes

We recommend that eGFR is used, preferably using the more accurate serum creatinine–based CKD Epidemiology Collaboration equation, without adjustment for race or ethnicity, when determining if certain therapies can be used to adjust medication dosages in diabetes.^16^^,^^17^ It is important to recognize that eGFR equations have several limitations,^18^ and hence an individualized pragmatic approach needs to be taken when deciding to initiate or cease medications solely on the basis of kidney function because of these limitations. In the clinical scenarios where creatinine-based eGFR equations may be less reliable (e.g., extremes of body weight) we would recommend using cystatin-based eGFR equations as a more accurate eGFR will better guide or determine treatment adjustments or changes in dosing.

### Glucose-Lowering Therapies for People who Have T2DM and CKD

The selection of individual classes of drugs tailored to the comorbidities that are frequently seen alongside CKD, will influence therapy selection (Figure 1 and Table 2). In addition, judicious combinations of different classes of drugs would need consideration. Although these guidelines focus on individual classes of glucose-lowering drugs, combinations of different classes are frequently prescribed to people with CKD. There is a relative dearth of studies providing high-quality evidence that specifically evaluate different drug combinations in people with T2DM and CKD, and this is clearly an area for both further research and clinical audit.

**Figure 1:**
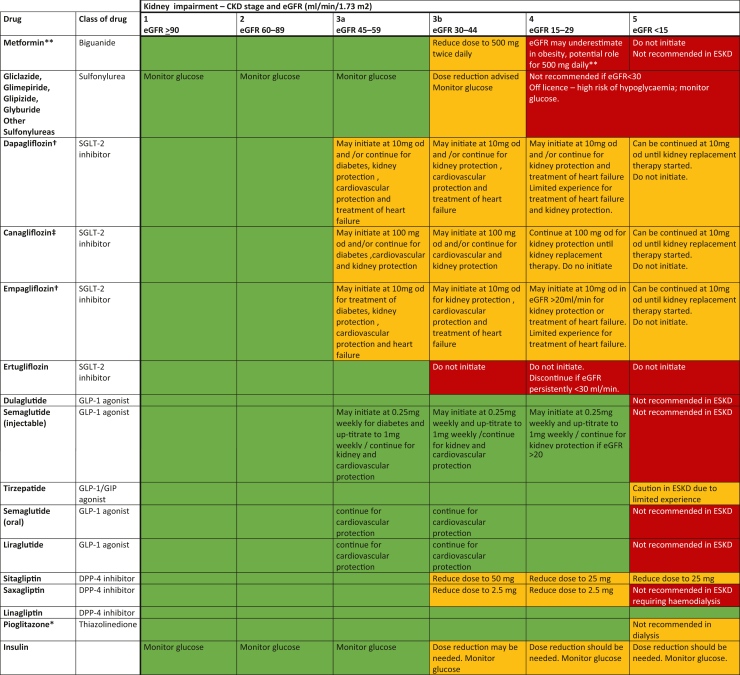
Glucose-lowering therapies—current licensing indications based on eGFR and cardio-kidney protection. Note that sick day guidance applies to metformin, all SGLT-2 inhibitors, and GLP-1 agonists. ∗Monitor for fluid retention; contraindicated in heart failure and macular edema. ∗∗CrCl and cystatin C may be used as an estimate of glomerular filtration rate to help clinical decision making (CrCl calculated using Cockcroft–Gault equation). †Dapagliflozin and Empagliflozin† can be initiated and continued for treatment of heart failure without reference to kidney function but no current evidence for initiation if eGFR < 20. ‡Canagliflozin can be initiated for kidney protection down to an eGFR of 30 ml/min per 1.73 m2 and be continued thereafter until the onset of dialysis or transplantation. Dapagliflozin† and Empagliflozin† can be initiated for kidney protection down to an eGFR of 15 ml/min per 1.73 m2 and be continued thereafter until the onset of dialysis or transplantation. CrCl, creatinine clearance; eGFR, estimated glomerular filtration rate; GLP-1, glucagon-like peptide 1; SGLT-2, sodium-glucose cotransporter 2.

**Table 2:** Relative and absolute contraindications to the selection of blood glucose-lowering therapies in people with T2DM and CKD

Condition	Drug	Note
Retinopathy	Pioglitazone	Absolute contraindication in diabetic maculopathy
	Semaglutide	Relative contraindication in people with marked hyperglycemia (HbA1c > 91 mmol/mol (10.5%) who have diabetic retinopathy requiring active ophthalmology treatment or follow-up: caution is advised
Bone health	Pioglitazone	Absolute contraindication in people who have had previous osteoporotic fractures; or relative contraindication in those with postmenopausal osteoporosis with neuropathy
	Canagliflozin	Relative contraindication in people with established osteoporotic fractures.
Foot health	SGLT-2 inhibitors	Absolute contraindication if a person has severe active diabetic foot disease with vascular complications or severe sepsis.
Heart failure	Pioglitazone	Absolute contraindication in people with established treated heart failure and where at-risk people have a raised natriuretic peptides or symptoms suggestive of heart failure
	Saxagliptin	Absolute contraindication in people with treated established heart failure
Pancreatic health	GLP-1 analogues and GLP-1 RA /GIP dual agonists	Absolute contraindication of GLP-1 analogues where an individual has previously documented pancreatitis; relative contraindication in people who are at risk of pancreatitis with raised triglycerides, those on steroid therapy, those using other drugs that are associated with pancreatitis or those with documented alcoholism
Bladder health	SGLT-2 inhibitors	Relative contraindication of all medications in this class in people who have documented neuropathic bladder and recurrent urinary infections
	Pioglitazone	Bladder cancer—contraindication to continuation or starting pioglitazone
Biliary tract health	GLP-1 analogues and GLP-1–GIP dual RAs	Relative contraindication if a person has active gall bladder disease

CKD, chronic kidney disease; HbA1c, glycated hemoglobin; GIP, glucose-dependent insulinotropic polypeptide; GLP-1, glucagon-like peptide 1; RA, receptor agonist; SGLT-2, sodium-glucose cotransporter 2, T2DM, type 2 diabetes mellitus.

#### Recommendations

1. Individualized HbA1c targets should be applied in the management of people with CKD, using the levels suggested in Table 1 (Grade 1B).
2. Additional comorbidities, that are frequently seen alongside CKD, and risk of hypoglycemia should influence therapy selection and HbA1c targets. In people who progress to advanced stages of CKD (eGFR < 45 ml/min) or those with rapid progression of CKD, more frequent monitoring of HbA1c and kidney function may be required. (Table 1, Table 2, and Figure 1) (Grade 1B).
3. Combinations of different classes of drugs are often needed to manage glycemia in CKD; judicious combinations of different classes should be considered based on their benefits and harms (Grade 2D).

### Insulin Therapy in People With CKD

#### Recommendations

1. There is no firm evidence that insulin therapy reduces the risk of progressive kidney disease. Therefore, the aim of insulin therapy should be to improve glycemic control and improve quality of life, with a low risk of hypoglycemia (Grade 1C).
2. Insulin requirements are likely to rise in the early stages of CKD because of increased insulin resistance (Grade 1C).
3. As eGFR declines, insulin requirements are likely to diminish through reduced kidney insulin clearance. In people with CKD stage 3b and below who are on insulin, and whose HbA1c is ≤ 58 mmol/mol (7.5%), reduction of insulin dose should be considered (Grade 1C).
4. People with CKD who are treated with insulin should undertake regular glucose monitoring (Grade 1C).
5. In people who are less able to manage with the requirements of a basal bolus regime, once daily regimes with longer-acting insulins should be considered (Grade 1D).
6. If people experience hypoglycemia on neutral protamine Hagedorn (NPH) insulin or premixed insulins, conversion to analogue insulins may be of benefit (Grade 1C).

### Metformin

The dose of metformin should be decreased to 500 mg twice a day if eGFR is < 45 ml/minute per 1.73 m^2^ and omitted if eGFR is < 30 ml/min per 1.73 m^2^. However, as stated previously, limitations of eGFR measurements need to be appreciated especially in those at extremes of body weight. Treatment should be paused in people at risk of tissue hypoxia or sudden deterioration in kidney function; for example, dehydration, severe infection, shock, sepsis, acute heart failure, respiratory failure, hepatic impairment, or those who have recently had a myocardial infarction.^19^

For most people, the benefits of metformin greatly outweigh the very small lactic acidosis risk. There may be subgroups of people who are at higher risk of lactic acidosis (not just because of impaired kidney function); however, the practical advice for clinicians and people contained in Table 3 and Figure 1 is relevant and in general supports the ongoing use of metformin for people with stable CKD stage 3.

**Table 3:** Action to be taken for selected medications when treating people with type 2 diabetes mellitus and CKD

**Metformin**	

**eGFR level**	**Action to be taken**

For all	• Practitioners have to weigh up the glycemic and cardiovascular benefits against the rare risk of associated lactic acidosis.
> 60 ml/min per 1.73 m^2^	• No kidney contraindication to metformin. • Some of these people are at increased risk because of other risk factors (see advice for increased vigilance groups in the bottom row of this table).
45–60 ml/min per 1.73 m^2^	• Continue use in people who were established on metformin; however, review the dose in light of glycemic control needs (maximum dose 2000 mg/d). • For new individuals who have no major active comorbidities, metformin commencement can be considered if age-related life expectancy is normal and vascular/diabetes risks are present. • Increase monitoring of kidney function (to every 3–6 mo).
30–45 ml/min per 1.73 m^2^	• Continue or commence with caution and explain the risks and benefits to the person. • Use lowest dose that achieves glycemic control (suggest a 50% dose up to 1000 mg/d). • Closely monitor kidney function (every 3 mo).
< 30 ml/min per 1.73 m^2^	• At this level of kidney function, we cannot give firm recommendations about the ongoing use of metformin. • Some specialists may choose to use metformin in selected people where they see that the benefits outweigh the risks. • Pharmacokinetic work would suggest that if metformin is used, a dose of 500–1,000 mg/d would result in 95% of people having peak metformin concentrations < 5 mg/l
Dialysis	• No current role
AKI (or at risk of AKI)	Review and consider (temporarily) pausing^a^ metformin in those who: • have acute changes in kidney function (a fall in eGFR of > 10 ml/min per 1.73 m^2^ over a period of days or weeks) • are at risk of AKI such as: o acute volume depletion and dehydration e.g., gastrointestinal upset, stomas, change in diuretic dose o during operative procedures with a high risk of hypotension or volume depletion o in the presence of hypotension or shock, e.g., severe infection o intravascular administration of iodinated contrast drugs (stop metformin on the day of and 2 d after X-ray–related i.v.contrast use) o coadministration with nephrotoxic drugs, e.g., nonsteroidal antiinflammatory drugs (NSAIDs) o those with acute illness who are also on drugs that are known precipitants of AKI in association with any angiotensin-converting enzyme (ACE) inhibitors or angiotensin receptor blockers (ARBs), and NSAIDs, especially combined with diuretics • those with previous episodes of AKI.
Recovery from AKI	• Once urine flow has returned to normal and GFR is > 30 ml/min per 1.73 m^2^, resume metformin at a low dose (e.g., 500–1,000 mg/d). • Monitor glucose control in outpatients and primary care before considering the further need for increasing doses.
Increased vigilance	Increased vigilance is needed for the following groups of people who are likely to be at a higher risk of lactic acidosis even with normal kidney function: • those with decompensated cardiac or respiratory failure • those with acute conditions that may cause tissue hypoxia, e.g., recent myocardial infarction (MI) or shock • those with hepatic insufficiency, acute alcohol intoxication or alcoholism.
**GLP-1 receptor agonists (GLP-1 RA): exenatide (Byetta and Bydureon), liraglutide, lixisenatide, dulaglutide, semaglutide, dual GLP-1 RA and GIP agonist, tirzepatide**	
eGFR level	Action to be taken
For all	• Older people: No dose adjustment is required based on age. Therapeutic experience in people aged ≥ 75 yrs is limited • Pediatric population: The safety and efficacy in children aged up to 18 yrs have not yet been established. No data are available. • Should not be used in people with type 1 diabetes mellitus or for the treatment of diabetic ketoacidosis. • No experience in those with congestive heart failure NYHA class IV and, therefore, not recommended in these people. • If pancreatitis is suspected, drug should be discontinued; if confirmed, then should not be restarted. Caution should be exercised in people with a history of pancreatitis.
> 60 ml/min per 1.73 m^2^	• No kidney contraindication to initiation or continuation. • Semaglutide injectable may be commenced for glucose lowering and kidney protection.
45–60 ml/min per 1.73 m^2^	• No kidney contraindication to initiation or continuation. • Semaglutide injectable may be commenced for glucose lowering and kidney protection.
30–45 ml/min per 1.73 m^2^	• Byetta and lixisenatide to be used “with caution” in people with creatinine clearance 30–50 ml/min, Bydureon should be stopped. • Liraglutide, dulaglutide, tirzepatide, and semaglutide have no kidney contraindication to initiation or continuation at standard doses. • Semaglutide injectable may be commenced for glucose lowering and kidney protection.
< 30 ml/min per 1.73 m^2^	• Liraglutide, dulaglutide, semaglutide, and tirzepatide have no kidney contraindication to initiation or continuation at standard doses. • Semaglutide injectable may be commenced for kidney protection. • Bydureon should be stopped
Dialysis	• No current role
AKI (or at risk of AKI)	Review and consider (temporarily) pausing^b^ in people who: • have acute changes in kidney function (a fall in eGFR of > 10 ml/min per 1.73 m^2^ over a period of days or weeks) • are at risk of AKI such as: o acute volume depletion and dehydration e.g., gastrointestinal upset, stomas, change in diuretic dose o operative procedures with a high risk of hypotension or volume depletion o in the presence of hypotension or shock, e.g., severe infection • have had previous episodes of AKI.
**eGFR level**	**Action to be taken**

**DPP-4 inhibitors: vildagliptin, saxagliptin, sitagliptin, linagliptin, alogliptin**	
eGFR level	Action to be taken
For all	• Older people (≥ 65 yrs): In general, no dose adjustment is recommended based on age. • Pediatric population: The safety and efficacy of DPP-4 inhibitors in children aged 0 to < 18 yrs have not yet been established. No data are available. • Vildagliptin should not be used in hepatic impairment. No dose adjustments are needed for mild to moderate hepatic impairment with linagliptin, alogliptin, sitagliptin, or saxagliptin. Caution needs to be exercised with sitagliptin use in those with severe hepatic impairment. Alogliptin and saxagliptin are not recommended in severe hepatic impairment. Only linagliptin is licensed for use in severe hepatic impairment. • Acute pancreatitis: DPP-4 inhibitors are associated with risk of developing acute pancreatitis. Caution should be exercised in those with history of pancreatitis. If pancreatitis is confirmed, DPP4 inhibitors should not be restarted. • Heart failure: DPP-4 inhibitors do not increase risk of major CV events or risk of hospitalization for heart failure, except saxagliptin, which is contraindicated in heart failure.
> 60 ml/min per 1.73 m^2^	• No kidney contraindication to initiation or continuation.
45–60 ml/min per 1.73 m^2^	• eGFR < 50 ml/min per 1.73 m^2^, reduce dose of vildagliptin to 50 mg once/d and alogliptin to 12.5 mg/d • No dose reduction needed for linagliptin, sitagliptin, or saxagliptin.
30–45 ml/min per 1.73 m^2^	• Reduce dose of sitagliptin to 50 mg/d, vildagliptin to 50 mg once/d, alogliptin to 12.5 mg/d, and saxagliptin to 2.5 mg/d. • No dose reduction needed for linagliptin. • Vildagliptin has limited data and should be used with caution.
< 30 ml/min per 1.73 m^2^	• Reduce dose of sitagliptin to 25 mg/d, alogliptin to 6.25 mg/d and saxagliptin to 2.5 mg/d. • No dose reduction needed for linagliptin. • Vildagliptin has limited data and should be used with caution.
Dialysis	• Reduce dose of sitagliptin to 25 mg/d, and alogliptin to 6.25 mg/d. • No dose reduction needed for linagliptin. • Saxagliptin is not recommended. • Vildagliptin has limited data and should be used with caution.
**SGLT-2 inhibitors: canagliflozin, dapagliflozin, empagliflozin, ertugliflozin**	
eGFR level	Action to be taken
For all	• Older people (≥ 65 yrs): In general, no dose adjustment is recommended based on age. However, the increased risk of volume depletion in frail older people should be considered • Pediatric population: the safety and efficacy of dapagliflozin in children aged up to 18 yrs have not yet been established. No data are available. • Active foot disease (either ulceration with sepsis or ischemia) avoid initiation and withdraw if this occurs. • Diabetic ketoacidosis: permanently discontinue if people develop diabetic ketoacidosis related to treatment.
> 60 ml/min per 1.73 m^2^	• No kidney contraindication to initiation or continuation. • Canagliflozin 100 mg/d may be commenced for glucose lowering and kidney protection. Dose can be increased to 300 mg for additional glucose lowering. • Dapagliflozin 10 mg/d may be commenced/continued for glucose lowering, heart failure and kidney protection. • Empagliflozin 10 mg/d may be commenced/continued for glucose lowering, heart failure, and kidney protection. Dose can be increased to 25 mg for additional glucose lowering.
45–60 ml/min per 1.73 m^2^	• Canagliflozin 100 mg/d may be commenced/continued for glucose lowering and kidney protection. • Dapagliflozin 10 mg/d may be commenced/continued for glucose lowering, heart failure, and kidney protection. • Empagliflozin 10 mg/d may be commenced/continued for glucose lowering, heart failure, and kidney protection • For other drugs in this class (such as ertugliflozin), current UK license recommends against initiation (see recommendations). Continuation of medication should be at the lower dose for canagliflozin and empagliflozin.
30–45 ml/min per 1.73 m^2^	• Canagliflozin 100 mg/d may be commenced/continued for kidney protection. • Dapagliflozin 10 mg/d may be commenced/continued for kidney protection and heart failure. • Empagliflozin 10 mg/d may be commenced/continued for kidney protection and heart failure • For glucose lowering, current license recommends against initiation or continuation.
20–30 ml/min per 1.73 m^2^	• Canagliflozin 100 mg/d may be continued for kidney protection until dialysis or kidney transplantation. • Empagliflozin 10 mg/d may be commenced/continued for heart failure • Dapagliflozin 10 mg/d may be commenced/continued for heart failure. • Empagliflozin 10 mg/d may be commenced/continued for kidney protection until dialysis or kidney transplantation. • Dapagliflozin 10 mg/d may be commenced/continued for kidney protection until dialysis or kidney transplantation.
Dialysis	• No current role
AKI (or at risk of AKI)	Review and consider (temporarily) pausing^c^ in people who: • have acute major changes in kidney function (a fall in eGFR of > 10 ml/min per 1.73 m^2^ over a period of days or weeks)^c^ • are at risk of AKI such as: o acute volume depletion and dehydration e.g., gastrointestinal upset, stomas, change in diuretic dose o operative procedures with a high risk of hypotension or volume depletion o in the presence of hypotension or shock, e.g., severe infection • have had previous episodes of AKI.

AKI, acute kidney injury; DPP-4, dipeptidyl peptidase-4; eGFR, estimated glomerular filtration rate; GIP, glucose-dependent insulinotropic polypeptide; GLP-1, glucagon-like peptide 1; RA, receptor agonist; SGLT-2, sodium-glucose cotransporter 2.

a Duration of pausing metformin should be based on the likely period of risk. In general, it should be resumed at a low dose after discharge if suitable and no contra-indications.

b Duration of pausing GLP-1 RA, GLP-1 RA/GIP dual agonist should be based on the likely period of risk. Essential to restart medications promptly once person is stable clinically and potential risks resolving.

c Duration of pausing SGLT-2 inhibitor should be based on the likely period of risk. Essential to restart medications promptly once person is stable clinically and potential risks resolving.

#### Recommendations

1. Metformin can be used down to an eGFR of 30 ml/min per 1.73 m^2^. The dosage should be reduced to 500 mg twice a day when the eGFR falls below 45 ml/min per 1.73 m^2^ (Grade 1B).
2. Metformin should be paused during periods of acute illness, particularly when a person has acute kidney injury. Everyone who is treated with metformin should be given sick day guidance, which should be reiterated at every medication review (Grade 1B).
3. In people with eGFR < 60 ml/min per 1.73 m^2^, metformin should be withheld 24 hours before and up to 48 hours after any procedure that requires the use of radiographic contrast media (Grade 1B).

### SGLT-2 Inhibitors

This section summarizes recent outcome trials where kidney outcomes were assessed as primary end point in people with T2DM and CKD with SGLT-2 inhibitors. Three clinical trials (summarized in brief below) have consistently shown that SGLT-2 inhibition on top of standard-of-care at the time of the studies (RAS inhibition) significantly reduces the risk of progression of CKD and associated CVD in people with T2DM and CKD.

The Canagliflozin and Kidney Events in Diabetes with Established Nephropathy Clinical Evaluation (CREDENCE) study was the first study of an SGLT-2 inhibitor to have kidney outcomes in its primary composite end point.^20^ People with T2DM and albuminuric CKD were randomized to receive canagliflozin 100 mg once daily or placebo. All participants had an eGFR of 30 to < 90 ml/min per 1.73 m^2^, albuminuria (urine albumin-to-creatinine ratio [ACR] > 33.9 to 565 mg/mmol [> 300 to 5000 mg/g]) and received RAS blockade. Of the recruits, 60% had an eGFR of 30 to 60 ml/min per 1.73 m^2^. The primary end point was a composite of ESKD (dialysis, transplantation, or sustained eGFR < 15 ml/min per 1.73 m^2^), a doubling of the serum creatinine or death from kidney or cardiovascular causes.

The relative risk of the primary end point was significantly lower in the canagliflozin group with event rates of 43.2 versus 61.2 per 1000 patient-years (hazard ratio [HR]: 0.70; 95% confidence interval [CI]: 0.59–0.82; *P* = 0.00001). The relative risk of the kidney-specific composite of ESKD, doubling of the creatinine level, or death from kidney causes was lower by 34% (HR: 0.66; 95% CI: 0.53–0.81; *P* < 0.001) and ESKD was lower by 32% (HR: 0.68; 95% CI: 0.54–0.86; *P* = 0.002).^20^ Of note, in this high-risk population, there were no significant increases in rates of lower limb amputation or fracture.

The canagliflozin group had a lower risk of cardiovascular death, myocardial infarction, or stroke (HR: 0.80; 95% CI: 0.67–0.95; *P* = 0.01) and hospitalization for heart failure (HR: 0.61; 95% CI: 0.47–0.80; *P* < 0.001).

The Dapagliflozin and Prevention of Adverse Outcomes in CKD (DAPA-CKD) trial assessed the effect of dapagliflozin on kidney and cardiovascular events in people with CKD (both with and without T2DM).^21^ In this study, 4094 participants with an eGFR between 25 and 75 ml/min per 1.73 m^2^ and urine ACR of 22.6 to 565 mg/mmol (200–5000 mg/g) were randomized to receive dapagliflozin 10 mg once daily or placebo. The primary outcome was a composite of sustained decline in eGFR of at least 50%, ESKD, or death from kidney or cardiovascular causes. Over a median of 2.4 years, the primary outcome event occurred in 197 of 2152 participants (9.2%) in the dapagliflozin group and 312 of 2152 participants (14.5%) in the placebo group (HR: 0.61; 95% CI: 0.51–0.72; *P* < 0.001). The HR for the kidney composite of a sustained decline in eGFR of at least 50%, ESKD, or death from kidney causes was 0.56 (95% CI: 0.45–0.68; *P* < 0.001). The effects observed were similar in people with T2DM to those without.^21^ Similarly, the HR for the composite of death from cardiovascular causes or hospitalization for heart failure was HR of 0.71 (95% CI: 0.55–0.92; *P* = 0.009).

The Study of Heart and Kidney Protection With Empagliflozin trial included 6609 people with CKD with an eGFR of 20 to 45 ml/min per 1.73 m^2^ (irrespective of the presence of albuminuria) or 45 to 90 ml/min per 1.73 m^2^ with raised urine ACR >200 mg/g (22.6 mmol/mol) who were randomized to receive empagliflozin 10 mg once daily or placebo.^22^ The primary outcome was a composite of progression of kidney disease (defined as ESKD, a sustained decrease in eGFR to < 10 ml/min per 1.73 m^2^, a sustained decrease in eGFR of ≥ 40% from baseline, or death from renal causes) or death from cardiovascular causes. During a median of 2.0 years of follow-up, progression of kidney disease or death from cardiovascular causes occurred in 432 of 3304 patients (13.1%) in the empagliflozin group and in 558 of 3305 patients (16.9%) in the placebo group (HR: 0.72; 95% CI: 0.64–0.82; *P* < 0.001).^22^ All-cause hospitalization was significantly lower for the empagliflozin group than in the placebo group (HR: 0.86; 95% CI: 0.78–0.95; *P* = 0.003).

The recent posttrial follow-up reported that in 4891 people (74%) enrolled where the use of open-label SGLT-2 inhibitors was similar in the 2 groups (43% in the empagliflozin group and 40% in the placebo group during the posttrial period). The HR for a primary-outcome event was 0.87 (95% CI: 0.76–0.99), which confirms that there is a continued cardio-renal benefit of empagliflozin for up to 12 months postdiscontinuation, but importantly highlights the need for the therapy be persisted with to maintain the observed “on treatment” greater risk reduction and related benefit.^23^ A collaborative meta-analysis that integrated the kidney outcomes from large placebo-controlled trials of SGLT-2 inhibitors from the SGLT-2 inhibitor Meta-Analysis Cardio-Renal Trialists’ Consortium concluded that in addition to the established cardiovascular benefits of SGLT-2 inhibitors, clinical evidence support their use for modifying risk of CKD progression and acute kidney injury, in patients with T2DM at high cardiovascular risk, and in patients with CKD or heart failure, irrespective of diabetes status, primary kidney disease or baseline kidney function.^24^

### Practical Aspects of Using SGLT-2 Inhibitors in People With T2DM and CKD

The observed kidney and cardiovascular benefits of SGLT-2 inhibitors are independent of the HbA1c-lowering effects of these agents in people with T2DM.

In people with diabetes and eGFR < 45 ml/min per 1.73 m^2^, treatment with SGLT-2 inhibitors do not lower HbA1c significantly.^1^ An SGLT-2 inhibitor can be initiated for kidney protection above an eGFR > 20 ml/min per 1.73 m^2^; however, if further glucose lowering is required, adding another class of medications to optimize diabetes control is recommended.^1^

If dapagliflozin, canagliflozin, or empagliflozin is started for CKD, the medication can be continued until dialysis initiation or renal transplantation (Figure 1). Regardless of urine ACR, we recommend the initiation of dapagliflozin or empagliflozin for people with T2DM and CKD where eGFR > 20 ml/min per 1.73 m^2^.

Diabetic ketoacidosis secondary to SGLT-2 inhibitor is rare in T2DM, with reported incidence between 1 in 1000 to 1 in 10,000 people.^1^^,^^24^ SGLT-2 inhibitor–induced diabetic ketoacidosis can present with normoglycemia or moderately raised glucose levels. It is important for clinicians to be aware of this so that diagnosis is not missed. Fournier’s gangrene (necrotizing fasciitis of the genitalia or perineum) is a rare but potentially life-threatening condition that is associated with several predisposing risk factors such poorly controlled diabetes, chronic alcoholism, renal failure, liver cirrhosis, and obesity.^25^, ^26^, ^27^ Fournier’s gangrene affects predominantly men and may present as severe pain or tenderness, erythema, and swelling in the genital or perineal area, with fever or malaise.^25^ Postmarketing cases of Fournier’s gangrene have been reported to be associated with the use of SGLT-2 inhibitors.^26^^,^^27^ For example, in the UK data from 2012 to 2018 reported by the medicines health regulatory agency noted 6 events of Fournier’s gangrene (4 in men and 2 in women) in association with SGLT-2 inhibitors.^28^ This corresponds to a UK estimated exposure to SGLT-2 inhibitors of 548,565 patient-years of treatment. People taking SGLT-2 inhibitors should be advised to seek urgent medical attention if they experience severe pain, tenderness, erythema, or swelling in the genital or perineal area accompanied by fever or malaise. If Fournier’s gangrene is suspected**,** SGLT-2 inhibitor treatment should be stopped and treatment should be started urgently (including antibiotics and surgical debridement) as appropriate.

#### Recommendations

1. We recommend the consideration of starting SGLT-2 inhibitors in all individuals with T2DM and CKD with an eGFR > 20 ml/min per 1.73 m^2^ (Grade 1A).
2. Where individuals are already receiving treatment with insulin or sulfonylureas, a reduction in dose of these drugs should be considered in people with T2DM and CKD with eGFR > 45 ml/min per 1.73 m^2^ and HbA1c that is not very high (e.g., < 64 mmol/mol [8%]) so as to reduce the risk of hypoglycemia (Grade 1A).
3. The initiation of SGLT-2 inhibitors in people who have active foot disease (ulceration, infection, sepsis, and ischemia) should be avoided and these agents should be paused in people who develop active infected and/or vascular foot complications while on treatment. SGLT-2 inhibitors should only be reinstated after foot problems have fully resolved and following discussion with the multidisciplinary foot team (expert opinion, no high-grade evidence).
4. SGLT-2 inhibitors should be withdrawn in all people who develop diabetic ketoacidosis. However, if a definitive cause for diabetic ketoacidosis is identified (e.g., low-calorie diet, postoperative catabolic state, and inappropriate cessation of insulin) reinstatement of SGLT-2 inhibitor may be considered depending on careful assessment of the individualized risks and benefits by a diabetes specialist (expert opinion, no high-grade evidence).
5. We do not recommend routine assessment of kidney function (creatinine and/or eGFR) within 6 to 8 weeks of SGLT-2 initiation because there is likely to be a transient and physiological deterioration, and this is not a reason to withdraw the drug (expert opinion, no high-grade evidence).
6. We recommend that sick day guidance applies, during which SGLT-2 inhibitors should be temporarily paused in scenarios; for example, when people are unable to maintain any oral fluid or food intake for > 24 hours or in settings of acute medical emergencies requiring urgent hospitalization or before major surgery (expert opinion, no high-grade evidence). It is, however, essential that once the acute event has resolved and the person has recovered, the SGLT-2 inhibitor is promptly restarted.

### GLP-1 RAs and Dual GLP-1 RA and GIP Agonists

Systematic reviews and meta-analyses suggest a clear beneficial class effect of GLP-1 RAs on the risk of CVD and albuminuria reduction.^29^^,^^30^ Currently, there is 1 primary kidney end point study reported with this class of agent in 3533 participants with T2DM and CKD where the Effects of Semaglutide on CKD in Patients with Type 2 Diabetes trial (FLOW)^12^ was evaluated. In this study, participants with T2DM with eGFR of 50 to 75 ml/min per 1.73 m^2^ and a urinary ACR between 33.9 mg/mmol (> 300 mg/g) and 565 mg/mmol (< 5000 mg/g) or an eGFR of 25 to < 50 ml/min per 1.73 m^2^ and ACR between 11.3 mg/mmol and 565 mg/mmol) (100–5000 mg/g) were randomized to receive subcutaneous semaglutide at a dose of 1.0 mg weekly or placebo. The primary outcome was major kidney disease events, a composite of the onset of kidney failure (dialysis, transplantation, or an eGFR < 15 ml/min per 1.73 m^2^, at least a 50% reduction in the eGFR from baseline, or death from kidney-related or cardiovascular causes).

This trial demonstrated both renal benefits and CVD mortality benefits with a 24% relative risk reduction of the risk of a primary outcome of major kidney disease events in the semaglutide group than in the placebo group (331 vs. 410 first events; HR: 0.76; 95% CI: 0.66–0.88; *P* = 0.0003). The risk of major cardiovascular events was 18% lower (HR: 0.82; 95% CI: 0.68–0.98; *P* = 0.029), and the risk of death from any cause 20% lower (HR: 0.80; 95% CI: 0.67–0.95, *P* = 0.01) in the semaglutide group as compared with placebo.^12^

The dose of injectable semaglutide was up-titrated from a starting dose of 0.25 mg/wk to 1 mg/wk over 8 weeks (0.25–0.50 mg over weeks and 0.5–1.0 mg over further 4 weeks). Similar benefits for the composite of the kidney-specific components of the primary outcome (HR: 0.79; 95% CI: 0.66–0.94) and for death from cardiovascular causes (HR: 0.71; 95% CI: 0.56–0.89) were observed. These results were observed as compared with standard-of-care (placebo group), which was RAS blockade; and of note approximately 15.6% of the cohort were on SGLT-2 inhibition at baseline, and no major heterogeneity of treatment effect observed in those on this combination. This evidence for the beneficial impact of GLP-1 RAs on kidney outcomes has been observed in several studies where reduction in albuminuria and prevention of eGFR fall were reported when analyses as secondary or exploratory outcomes.^1^^,^^30^, ^31^, ^32^ Further primary kidney end point trials with other agents in this class of medications in people with CKD are ongoing and include promising data from the dual GLP-1 RAs and GIP agonist, tirzepatide.^33^ In Figure 1, we present the detailed information on frequently used GLP-1 RAs and GLP-1 RAs/GIP agonist dosing considerations in CKD.

#### Recommendations

1. We recommend the consideration of the injectable once weekly semaglutide (1 mg/wk subcutaneously) in people with T2DM and albuminuric CKD who have eGFR > 25 ml/min per 1.73 m^2^, irrespective of glycemic control (Grade 1B).
2. Other agents within the class of GLP-1 RA or GLP-1 RA /GIP RA dual agonists can be used for the improvement of glycemic control with a low risk of both hypoglycemia and beneficial effects of weight loss in obese people with T2DM and CKD (Grade 1A).
3. There is evidence of protection from CVD with some GLP-1 RAs in people who have T2DM and a high risk of CVD (Grade 1A).
4. People with CKD who are treated with GLP-1 RAs or GLP-1 RA/GIP RA dual agonists need to only perform regular self-monitoring of blood glucose when they are also being treated with drugs that can cause hypoglycemia (sulfonylureas and insulins) (Grade 1A).
5. We recommend caution for the use of GLP-1 RA or GLP-1 RA/GIP RA dual agonists in people with CKD with advanced active diabetic retinopathy (proliferative diabetic retinopathy) and elevated HbA1c (>86 mmol/mol or 10%) and if treatment is started, avoidance of rapid reduction in HbA1c and liaising with ophthalmology teams to ensure retinal surveillance is in place (expert opinion no high grade evidence).

### DPP-4 Inhibitors

DPP-4 inhibitors bind selectively to DPP-4 and prevent the rapid hydrolysis GLP-1. They have a modest glucose-lowering effect, compared with other oral hypoglycemic agents. DPP-4 inhibitors are known to have a very low risk of hypoglycemia and are generally associated with a favorable safety and tolerability profile in people with T2DM and mild-to-severe kidney impairment (Figure 1).^34^

#### Recommendations

1. We recommend that people with CKD of all stages are suitable for treatment with DPP-4 inhibitors (Grade 1B).
2. We recommend that doses of DPP-4 inhibitors are appropriately reduced in accordance with the degree of kidney impairment (including maintenance hemodialysis) except linagliptin (Grade 1B).
3. People with CKD can be safely prescribed DPP-4 inhibitors without the risk of hypoglycemia or weight gain at all stages of kidney disease (Grade 1B).
4. There are no current data to suggest that DPP-4 inhibitors (except saxagliptin) are associated with an excess risk of hospitalization for heart failure (Grade 1A).

### Sulfonylureas

There is very little comparative randomized controlled trial evidence of the use of sulfonylureas in CKD. People with T2DM and CKD who are on sulfonylurea treatment are at increased risk of hypoglycemia. We therefore advise regular capillary blood glucose (CBG) monitoring for people with CKD on sulfonylurea treatment. All sulfonylureas should be avoided where possible in advanced CKD (stage 4 and 5). In Table 2, Table 3 and Figure 1, we present detailed information and practical clinical considerations when using sulfonylureas in CKD.

### Pioglitazone

Pioglitazone is one of the few oral glucose-lowering drugs that is licensed for use in people with eGFR < 30 ml/min per 1.73 m^2^. Pioglitazone should be avoided if there is evidence of heart failure or macular edema or osteoporosis. Because these comorbidities may be frequent in people with CKD, people should be carefully and regularly monitored for fluid retention or other adverse effects. More detailed information on the use of pioglitazone in CKD and related recommendations are presented in Table 2, Table 3 and Figure 1 and this recent review.^2^

### nsMRAs

Our main focus in this article was on pharmacological management of hyperglycemia in people with T2DM and CKD. However the emerging role of finerenone, an nsMRA is important to acknowledge because this class of medications have emerged as an additional pillar for the management of T2DM and CKD.^35^ A recent review highlights the clinical utility and consideration of starting nsMRA.^36^ Steroidal mineralocorticoid receptor antagonists such as spironolactone and eplerenone lower blood pressure, reduce albuminuria proteinuria, delay progression of CKD, and reduce mortality in heart failure.^36^^,^^37^ However, their use is often limited by their tendency to cause hyperkalemia, an effect which is pronounced in people with T2DM and CKD. Finerenone is an nsMRA with greater mineralocorticoid receptor affinity and selectivity.^36^ These characteristics of finerenone are associated with less hyperkalemia and minimal gynecomastia compared with the steroidal agents.^36^ Based on the strong evidence of cardio-renal protection offered by the addition of finerenone to RAS inhibition observed in recent randomized controlled trials, we suggest in people with T2DM and CKD who have persistent albuminuria (ACR > 30 mg/mmol) despite the use of maximum tolerated doses of RAS inhibition and SGLT-2 inhibitors, to consider addition of finerenone.^36^^,^^38^ Finerenone can be used if eGFR is ≥ 25 ml/min per 1.73 m^2^ and serum potassium is normal (< 5 mmol/l). It is important to monitor serum potassium level after commencing treatment and the dose of finerenone will need to be adjusted according to potassium levels. Further detailed guidance on the practical use of finerenone in people with T2DM and CKD is available in this recent recommendation^36^

### Cost-Effectiveness of Newer Treatments

CKD has a significant financial burden for many countries worldwide. The mean annual costs of CKD increases substantially with CKD stage, with a recent global study of 31 countries reporting annual costs for CKD stage 3A of US$3070, which increases significantly in stage 5 CKD (hemodialysis, $57,334; peritoneal dialysis, $49,490 with transplantation costs of $75,326 (incident) and $16,672 (ongoing)]^39^ The authors noted that these costs for CKD are much higher than for other diseases such as myocardial infarction ($18,294/yr) and heart failure ($8463/yr).^39^

In this context, there have been several systematic reviews as well as detailed cost effectiveness studies that have demonstrated the benefits of newer therapies for CKD.^40^, ^41^, ^42^, ^43^, ^44^ The National Institute for Health and Care Excellence, UK evidence review and related cost-effectiveness analyses demonstrated that SGLT-2 inhibitors were cost-effective in the UK health setting for people with T2DM and CKD, and these findings in part informed changes in national T2DM guidelines, where early adoption and consideration for this class of agent for cardio-renal protection independent of baseline HbA1c was proposed.^45^^,^^46^

However, as far as we are aware there are to date no high-quality cost effectiveness analyses or studies that specifically investigate dual or triple combination of newer treatments on top of background RAS inhibition with angiotensin-converting enzyme inhibitor or angiotensin receptor blocker. This lack of studies reflects the limited evidence and data available at present from randomized controlled trials or large registries that have specifically looked at benefits of multiple combination treatment in CKD.

Factors that influence cost-effectiveness and access include medication acquisition costs, cost of CKD and related comorbidity management (because people with longer survival will experience greater lifetime costs for CKD and associated comorbidities such as CVD).

Newer treatments for CKD offer significant promise and are generally cost effective but can be expensive with substantial budget or financial impact given the potentially large treatment population. However, cost-effectiveness will improve over time with increasing numbers of agents moving to generic compounds, which will lower medication costs, as well as competition which can further reduce costs.

The identification of many individuals with or at risk for CKD who may be suitable for newer medications can be overwhelming to many health care systems (private, out of pocket, insurance bases or public/state funded, or hybrid models of care) and related economics. Therefore, cost-effectiveness needs to be routinely incorporated into clinical guidelines to help guide optimal decisions because clear concise information on value-for-money and related improvement in health equity will help better guide and inform health care delivery.

### Conclusion

People with T2DM and CKD have increased risk of morbidity and mortality. Hyperglycemia is a modifiable risk factor for cardiovascular complications and progression of CKD. Individualized HbA1c targets should be applied in the management of people with CKD, using the levels suggested in this guidance.

Delaying ESKD and reducing CVD risk are essential to improve outcomes in this high-risk population. There is now conclusive evidence and consensus that SGLT-2 inhibitors significantly reduce progression of CKD and onset of ESKD in people with T2DM and CKD on top of standard care, which is RAS inhibition. More recently there is evidence for the use nsMRA and GLP-1 RA which can also delay progression of CKD, which has resulted in a concept of pillars of cardio-renal pharmacotherapy for CKD and thereby the potential for addressing residual cardio-renal risk with a multipronged approach with medications that act on distinct pathways to confer organ protection.^9^^,^^47^ Despite advances in the field, a major barrier is the lack of implementation and inequality of access or delivery of guideline-based interventions and treatments for people with T2DM and CKD.^48^ In view of this, we propose a tiered approach with addressing key foundational goals first to address cardio-renal disease in T2DM and CKD.^38^ Indeed because most people with T2DM will succumb to premature CVD before reaching ESKD, it is essential that a cardio-renal focus is emphasized from the outset.

In Figure 2, we highlight the tiered approach we propose to address burden of CKD progression as well as high CVD risk in people with T2DM and CKD. We acknowledge that there is currently limited randomized control trial data on the role of combination therapy or triple therapy with GLP-1 RA, SGLT-2 inhibitors, or nsMRA on reducing progression of kidney disease. The available data to date from secondary or subanalyses of trials, simulation type analyses from recent studies, and real world data suggest potential complementary and additive effects; and in our opinion, combination treatment of GLP-1 RA and SGLT-2 inhibitors should be considered early in the management of CKD in people with diabetes.^49^ Such a combination approach may aid cardio-renal risk reduction by targeting residual risk and improve glycemic control with low risk of hypoglycemia in people with T2DM and CKD. In people with residual albuminuria despite combination treatment with GLP-1 RA and SGLT-2 inhibitors, adding in a nsMRA in people at high risk of progression to kidney failure or with known heart failure may be appropriate and should be considered.

**Figure 2:**
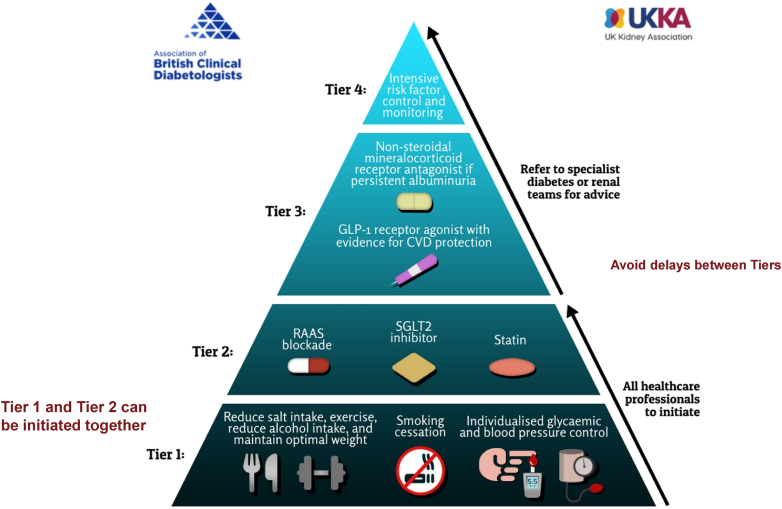
A tiered approach to managing people with type 2 diabetes mellitus and CKD. An overview of the joint Association of British Clinical Diabetologists and UK Kidney Association (ABCD-UKKA) guidelines. With permission from Dasgupta *et al.*^38^ ∗Individualized approach to intensive risk factor control and monitoring which can be based on factors such as life-time cardiovascular and renal risk, life expectancy, frailty, and resources. CKD, chronic kidney disease.

The mainstay of the pharmacological management of CKD in T2DM are RAS inhibition, SGLT-2 inhibition, GLP-1 RA, nsMRA, and lipid-lowering therapy in conjunction with lifestyle modification to reduce the progression of CKD and CVD risk. The management of people with T2DM and CKD require an individualized approach, where combination therapy of these key pillars of care will need to be used to address and mitigate residual risk of cardio-renal disease.

## Disclosure

SB reports receiving personal fees from Abbott, AstraZeneca, Boehringer Ingelheim, Eli Lilly, Merck Sharp & Dohme, Novo Nordisk, and Sanofi Aventis; and being a shareholder in Glycosmedia. DB reports receiving speaker fees from Vifor Pharma; honoraria for advisory board from Bayer and Medice; and research grant from AstraZeneca. ID reports receiving research grants from Medtronic and Sanofi-Genzyme, receiving honoraria for attending advisory board and speaker meetings from GlaxoSmithKline, AstraZeneca, and Sanofi-Genzyme; and being the national lead for 3 GSK trials. PD reports receiving honoraria for advisory work and/or lecture fees from AstraZeneca, Boehringer Ingelheim, Eli Lilly, Merck Sharp & Dohme, Napp Pharmaceuticals, Novo Nordisk, and Sanofi. JK reports receiving research grants from AstraZeneca and Sanofi; and receiving speaker fees and attending advisory boards from Boehringer Ingelheim, AstraZeneca, Sanofi, and Napp. KM reports receiving speaker fees and attending advisory board from Vifor, AstraZeneca, Bayer, Boehringer Ingelheim, Pharmacomsos, Napp, and Vifor Fresenius; and receiving a grant from AstraZeneca. PW reports receiving honoraria for delivering educational meetings and/or attending advisory boards for Abbott, AstraZeneca, Bayer, Boehringer Ingelheim, Eli Lilly, Merck Sharp & Dohme, Napp, Sanofi, Novo, and Vifor Pharmaceuticals. NM reports receiving honoraria for delivering educational meetings and/or attending advisory boards and conferences for Abbott, AstraZeneca, Bayer, Boehringer Ingelheim, Eli Lilly, Menarini, Novo Nordisk, and Roche. NK Received honoraria and travel grants from Abbott, AZ, Bayer, BI, Daiichi-Sankyo, Lilly, Sanofi and Menarini. All the other authors declared no competing interests.

## Supplementary Material

Supplementary File (PDF) **Figure S1.** Evidence grades for the recommendations.

## References

[1] Rossing P., Caramori M.L., Chan J.C.N. (2022). Executive summary of the KDIGO 2022 Clinical Practice Guideline for Diabetes Management in Chronic Kidney Disease: an update based on rapidly emerging new evidence. Kidney Int.

[2] Karalliedde J., Winocour P., Chowdhury T.A. (2022). Clinical practice guidelines for management of hyperglycaemia in adults with diabetic kidney disease. Diabet Med.

[3] Lewis E.J., Hunsicker L.G., Bain R.P., Rohde R.D. (1993). The effect of angiotensin-converting-enzyme inhibition on diabetic nephropathy. The collaborative study group. N Engl J Med.

[4] Chaturvedi N. (1997). Randomised placebo-controlled trial of lisinopril in normotensive patients with insulin-dependent diabetes and normoalbuminuria or microalbuminuria. The EUCLID Study Group. Lancet.

[5] Brenner B.M., Cooper M.E., de Zeeuw D. (2001). Effects of losartan on renal and cardiovascular outcomes in patients with type 2 diabetes and nephropathy. N Engl J Med.

[6] Lewis E.J., Hunsicker L.G., Clarke W.R. (2001). Renoprotective effect of the angiotensin-receptor antagonist irbesartan in patients with nephropathy due to type 2 diabetes. N Engl J Med.

[7] Sica D.A., Bakris G.L. (2002). Type 2 diabetes: RENAAL and IDNT--the emergence of new treatment options. J Clin Hypertens (Greenwich).

[8] Chaudhuri A., Ghanim H., Arora P. (2022). Improving the residual risk of renal and cardiovascular outcomes in diabetic kidney disease: a review of pathophysiology, mechanisms, and evidence from recent trials. Diabetes Obes Metab.

[9] Chaudhry K., Karalliedde J. (2024). Chronic kidney disease in type 2 diabetes: the size of the problem, addressing residual renal risk and what we have learned from the CREDENCE trial. Diabetes Obes Metab.

[10] Brown E., Wilding J.P.H., Alam U., Barber T.M., Karalliedde J., Cuthbertson D.J. (2021). The expanding role of SGLT2 inhibitors beyond glucose-lowering to cardiorenal protection. Ann Med.

[11] Kidney Disease: Improving Global Outcomes (KDIGO) CKD Work Group (2024). KDIGO 2024clinical practice guideline for the evaluation and management of chronic kidney disease. Kidney Int.

[12] Perkovic V., Tuttle K.R., Rossing P. (2024). Effects of semaglutide on chronic kidney disease in patients with type 2 diabetes. N Engl J Med.

[13] American Diabetes Association Professional Practice Committee (2024). 9. Pharmacologic approaches to glycemic treatment: standards of care in Diabetes-2024. Diabetes Care.

[14] Wijewickrama P., Williams J., Bain S. (2023). Narrative review of glycemic management in people with diabetes on peritoneal dialysis. Kidney Int Rep.

[15] Frankel A.H., Wahba M., Ashworth V. (2023). Management of adults with diabetes on dialysis: summary of recommendations of the Joint British Diabetes Societies guidelines 2022. Diabet Med.

[16] Wang Y., Katzmarzyk P.T., Horswell R., Zhao W., Johnson J., Hu G. (2016). Comparison of the heart failure risk stratification performance of the CKD-EPI equation and the MDRD equation for estimated glomerular filtration rate in patients with type 2 diabetes. Diabet Med.

[17] Delgado C., Baweja M., Crews D.C. (2021). A unifying approach for GFR estimation: recommendations of the NKF-ASN task force on reassessing the inclusion of race in diagnosing kidney disease. J Am Soc Nephrol.

[18] Nair S., Mishra V., Hayden K. (2011). The four-variable modification of diet in renal disease formula underestimates glomerular filtration rate in obese type 2 diabetic individuals with chronic kidney disease. Diabetologia.

[19] Home P., Mant J., Diaz J., Turner C., Guideline Development Group (2008). Management of type 2 diabetes: summary of updated NICE guidance. BMJ.

[20] Perkovic V., Jardine M.J., Neal B. (2019). Canagliflozin and renal outcomes in type 2 diabetes and nephropathy. N Engl J Med.

[21] Heerspink H.J.L., Stefánsson B.V., Correa-Rotter R. (2020). Dapagliflozin in patients with chronic kidney disease. N Engl J Med.

[22] Herrington W.G., Staplin N., Wanner C. (2023). Empagliflozin in patients with chronic kidney disease. N Engl J Med.

[23] EMPA-KIDNEY Collaborative Group, Herrington W.G., Staplin N. (2025). Long-term effects of empagliflozin in patients with chronic kidney disease. N Engl J Med.

[24] Nuffield Department of Population Health Renal Studies Group (2022). SGLT2 inhibitor Meta-Analysis Cardio-Renal Trialists’ Consortium. Impact of diabetes on the effects of sodium glucose co-transporter-2 inhibitors on kidney outcomes: collaborative meta-analysis of large placebo-controlled trials. Lancet.

[25] Eke N. (2000). Fournier’s gangrene: a review of 1726 cases. Br J Surg.

[26] Silverii G.A., Dicembrini I., Monami M., Mannucci E. (2020). Fournier’s gangrene and sodium-glucose co-transporter-2 inhibitors: a meta-analysis of randomized controlled trials. Diabetes Obes Metab.

[27] Wang T., Patel S.M., Hickman A. (2020). SGLT2 inhibitors and the risk of hospitalization for Fournier’s gangrene: a nested case-control study. Diabetes Ther.

[28] The Medicines and Healthcare products Regulatory Agency (2019). Drug Safety Update. Department of Health and Social Care.

[29] Palmer S.C., Tendal B., Mustafa R.A. (2021). Sodium-glucose cotransporter protein-2 (SGLT-2) inhibitors and glucagon-like peptide-1 (GLP-1) receptor agonists for type 2 diabetes: systematic review and network meta-analysis of randomised controlled trials. BMJ.

[30] Sattar N., Lee M.M.Y., Kristensen S.L. (2021). Cardiovascular, mortality, and kidney outcomes with GLP-1 receptor agonists in patients with type 2 diabetes: a systematic review and meta-analysis of randomised trials. Lancet Diabetes Endocrinol.

[31] Kristensen S.L., Rørth R., Jhund P.S. (2019). Cardiovascular, mortality, and kidney outcomes with GLP-1 receptor agonists in patients with type 2 diabetes: a systematic review and meta-analysis of cardiovascular outcome trials. Lancet Diabetes Endocrinol.

[32] Bosch C., Carriazo S., Soler M.J., Ortiz A., Fernandez-Fernandez B. (2023). Tirzepatide and prevention of chronic kidney disease. Clin Kidney J.

[33] Heerspink H.J.L., Sattar N., Pavo I. (2022). Effects of tirzepatide versus insulin glargine on kidney outcomes in type 2 diabetes in the SURPASS-4 trial: post-hoc analysis of an open-label, randomised, phase 3 trial. Lancet Diabetes Endocrinol.

[34] Monami M., Dicembrini I., Martelli D., Mannucci E. (2011). Safety of dipeptidyl peptidase-4 inhibitors: a meta-analysis of randomized clinical trials. Curr Med Res Opin.

[35] Bakris G.L., Agarwal R., Anker S.D. (2020). Effect of finerenone on chronic kidney disease outcomes in type 2 diabetes. N Engl J Med.

[36] De P., Khine M.T., Frankel A. (2025). Finerenone in the management of diabetes kidney disease. BMC Nephrol.

[37] Ocello A., La Rosa S., Fiorini F. (2019). Antifibrotic renal role of mineralcorticoid receptor antagonists. G Ital Nefrol.

[38] Dasgupta I., Zac-Varghese S., Chaudhry K. (2024). Current management of chronic kidney disease in type-2 diabetes-A tiered approach: an overview of the joint Association of British Clinical Diabetologists and Uk Kidney association (ABCD-UKKA) guidelines. Diabet Med.

[39] Jha V., Al-Ghamdi S.M.G., Li G. (2023). Global economic burden associated with chronic kidney disease: a pragmatic review of medical costs for the inside CKD research programme. Adv Ther.

[40] Yoshida Y., Cheng X., Shao H., Fonseca V.A., Shi L. (2020). A systematic review of cost-effectiveness of sodium-glucose cotransporter inhibitors for type 2 diabetes. Curr Diab Rep.

[41] McEwan P., Darlington O., Miller R. (2022). Cost-effectiveness of dapagliflozin as a treatment for chronic kidney disease: A health-economic analysis of DAPA-CKD. Clin J Am Soc Nephrol.

[42] Ramos M., Gerlier L., Uster A., Muttram L., Frankel A.H., Lamotte M. (2024). Cost-effectiveness of empagliflozin as add-on to standard of care for chronic kidney disease management in the United Kingdom. J Med Econ.

[43] Zheng C., Wu J., Li N. (2025). Cost-effectiveness of finerenone added to standard of care for patients with type 2 diabetes-related chronic kidney disease in the United States. Diabetes Obes Metab.

[44] Morton J.I., Marquina C., Shaw J.E. (2023). Projecting the incidence and costs of major cardiovascular and kidney complications of type 2 diabetes with widespread SGLT2i and GLP-1 RA use: a cost-effectiveness analysis. Diabetologia.

[45] NICE Evidence Reviews Collection (2021). https://www.ncbi.nlm.nih.gov/books/NBK591834/.

[46] Moran G.M., Bakhai C., Song S.H., Agwu J.C. (2022). Guideline Committee. Type 2 diabetes: summary of updated NICE guidance. BMJ.

[47] Kearney J., Gnudi L. (2023). The pillars for renal disease treatment in patients with type 2 diabetes. Pharmaceutics.

[48] Phillips K., Hazlehurst J.M., Sheppard C. (2024). Inequalities in the management of diabetic kidney disease in UK primary care: a cross-sectional analysis of a large primary care database. Diabet Med.

[49] Neuen B.L., Heerspink H.J.L., Vart P. (2024). Estimated lifetime cardiovascular, kidney, and mortality benefits of combination treatment with SGLT2 inhibitors, GLP-1 receptor agonists, and nonsteroidal MRA compared with conventional care in patients with type 2 diabetes and albuminuria. Circulation.

