# Supplementary material for: Association of British Clinical Diabetologists and UK Kidney Association Joint Clinical Practice Guidelines for the Pharmacological Management of Hyperglycemia in Adults With Type 2 Diabetes Mellitus and CKD
Source: Kidney Int Rep. 2025 Jul 31;10(10):3318–31. doi: 10.1016/j.ekir.2025.07.028 (PMC12545811; doi:10.1016/j.ekir.2025.07.028)
Supplement: Supplementary File (PDF) — Figure S1. Evidence grades for the recommendations. [file mmc1.pdf]

Supplement file 1

Evidence grades for the recommendations

The following evidence grading has been used to determine the strength of the recommendations

1A – Strong recommendation: high-quality evidence

1B – Strong recommendation: moderate-quality evidence

1C – Strong recommendation: low-quality evidence

1D – Strong recommendation: very low-quality evidence

2A – Weak recommendation: high-quality evidence

2B – Weak recommendation: moderate-quality evidence

2C – Weak recommendation: low-quality evidence

2D – Weak recommendation: very low-quality evidence
